# Radiomics-Based Artificial Intelligence Differentiation of Neurodegenerative Diseases with Reference to the Volumetry

**DOI:** 10.3390/life12040514

**Published:** 2022-03-31

**Authors:** Eva Y. W. Cheung, Anson C. M. Chau, Fuk Hay Tang, on behalf of the Alzheimer’s Disease Neuroimaging Initiative

**Affiliations:** 1 School of Medical Health and Sciences, Tung Wah College, 19/F, 31 Wylie Road, Ho Man Tin, Hong Kong, China; cheungevayw@gmail.com or; 2 Medical Radiation Science, Allied Health and Human Performance Unit, University of South Australia, City East Campus, Bonython Jubilee Building, 1-26, Adelaide, SA 5001, Australia; anson.chau@unisa.edu.au

**Keywords:** Alzheimer’s Disease, mild cognitive impairment, radiomics, volumetry, Artificial Intelligence, machine learning, random forest

## Abstract

This study aimed to build automated detection models—one by brain regional volume (V-model), and the other by radiomics features of the whole brain (R-model)—to differentiate mild cognitive impairment (MCI) from cognitive normal (CN), and Alzheimer’s Disease (AD) from mild cognitive impairment (MCI). The objectives are to compare the models and identify whether radiomics or volumetry can provide a better prediction for differentiating different types of dementia. Method: 582 MRI T1-weighted images were retrieved from the Alzheimer’s Disease Neuroimaging Initiative (ADNI) database, which is a multicenter operating open source database for AD. In total, 97 images of AD, 293 images of MCI patient and 192 images of cognitive normal were divided into a training, a validation and a test group at a ratio of 70:15:15. For each T1-weighted image, volumetric segmentation was performed with the image analysis software FreeSurfer, and radiomics features were retrieved by imaging research software 3D slicers. Brain regional volume and radiomics features were used to build the V-model and R-model, respectively, using the random forest algorithm by R. The receiver operating characteristics (ROC) curve of both models were used to evaluate their diagnostic accuracy and reliability to differentiate AD, MCI and CN. Results: To differentiate MCI and CN, both V-model and R-model achieved excellent performance, with an AUC of 0.9992 ± 0.0022 and 0.9850 ± 0.0032, respectively. No significant difference was found between the two AUCs, indicating both models attained similar good performance. In MCI and AD differentiation, the V-model and R-model yielded AUC of 0.9986 ± 0.0013 and 0.9714 ± 0.0175, respectively. The best performance was to differentiate AD from CN, where the V-model and R-model yielded AUC of 0.9994 ± 0.0019 and 0.9830 ± 0.009, respectively. The results suggested that both volumetry and radiomics approaches could be used in differentiating AD, MCI and CN, based on T1 weighted MR images using random forest algorithm successfully. Conclusion: This study showed that the radiomics features from T1-weighted MR images achieved excellence performance in differentiating AD, MCI and CN. Compared to the volumetry method, the accuracy, sensitivity and specificity are slightly lower in using radiomics, but still attained very good and reliable classification of the three stages of neurodegenerations. In view of the convenience and operator independence in feature extraction, radiomics can be a quantitative biomarker to differentiate the disease groups.

## 1. Introduction

In the past decades, structural MRI has been used to aid the diagnosis of AD, MCI and other forms of dementia [1,2,3]. Many studies demonstrated that when compared to cognitive normal (CN), patients with AD have a significant reduction in the volume of medial temporal lobe, including the hippocampus, entorhinal cortex, parahippocampal gyrus, subiculum and amygdala [4,5]. Through quantitative measurement of volume of regions-of-interest (ROI) in hippocampus, it is suggested that the hippocampal volume reduction can be a diagnostic factor for AD, and the rate of reduction can be an indicator to predict the disease progression [6,7]. However, for the prodromal stage of AD, the volume of other brain regions that relevant to AD or MCI, require extensive knowledge and experience from clinicians, to identify the regions of interest for staging AD or MCI. In addition, brain regional volume change is minimal and cannot be detected easily at the stage of disease. There is an urge to identify other biomarkers, which can be used to aid early detection for neurodegenerative disease.

In structural MRI, brain regional volume could be quantified by voxel-based morphometry (VBM) with DARTEL algorithm [8,9]. In recent years, the surface-based segmentation, which suggested by FreeSurfer was widely used. It is a software package for the analysis of structural neuroimaging data with whole brain segmentation capabilities [10]. It allows reconstruction of cortical surface models, labelling of regions on both cortical surface and subcortical structures. Additionally, it allows for hippocampal volume assessment [11,12], connectivity-based segmentation [13] and sub-regional corpus callosum segmentation [14]. The surface-based method is sensitive for evaluating cortical atrophy and subcortical brain regional volume change, which have been employed extensively to investigate in aid in staging AD, MCI and CN.

In recent years, radiomics analysis has been widely applied in medical imaging. It is a novel technique to identify local image features, such as gray level invariant features (GLIF) that related in the detection and quantification of dementia. Previous studies showed that combination of cortical thickness and hippocampal texture aided the diagnosis of AD, MCI and CN [15]. Another study showed that AD, MCI and CN showed different 3D texture features in structural images [16]. Nowadays, other radiomics features including different types of grey level matrixes are available to be extracted from T1 weighted MR image. Deep radiomics analysis of image features may aid in characterize local texture and global measurements in order to discriminate AD, MCI and CN in different perspectives.

In this study, we aimed at developing a computer assisted model to differentiate AD, MCI and CN using radiomics features. Additionally, we compared its prediction performance with another model which developed based on brain regional volume, to assess whether using volumetric analysis or image features from structural image (radiomics) can provide a more accurate differentiation of different types of dementia, including AD, MCI, when compared to CN.

## 2. Material and Methods

### 2.1. Subjects

Data used in the preparation of this article were obtained from the Alzheimer’s Disease Neuroimaging Initiative (ADNI) database (adni.loni.usc.edu). The ADNI was launched in 2003 as a public–private partnership, led by Principal Investigator Michael W. Weiner, MD. The primary goal of ADNI has been to test whether serial magnetic resonance imaging (MRI), positron emission tomography (PET), other biological markers, and clinical and neuropsychological assessment can be combined to measure the progression of mild cognitive impairment (MCI) and early Alzheimer’s Disease (AD). For up-to-date information, see www.adni-info.org, accessed on 22 January 2022. The use of the ADNI data was approved by the institutional review board at each site, and all the participants provided their written consent. In this study, a total of 582 T1 MPRAGE images were collected from ADNI1 cohorts from ADNI database. All eligible participants underwent brain MRI scanning and clinical cognitive evaluations, and were clinically followed-up for more than 36 months. There were 192 images of CN, 293 images of MCI and 97 image of AD. All images were randomly divided into a training group, validation group and test group in ratio 70:15:15.

### 2.2. Image Acquisition

Regarding to the ADNI data, The MR T1-weighted structural images were acquired using Siemens scanner, using a 3D MPRAGE (magnetization prepared rapid gradient-echo imaging) sequence. The scanning parameters were listed in the Supplementary file and is available on the ADNI website [2].

### 2.3. Preprocessing of Images

After the T1-weighted MPRAGE MR images in Digital Imaging and Communications In Medicine (DICOM) format were downloaded from the ADNI, all images were standardized to minimize the textural features discrepancy between images. Firstly, each T1-weighted MPRAGE MR image was converted to Neuroimaging Informatics Technology Initiative (NIFTI) format. Secondly, intensity normalization and bias field inhomogeneity correction were performed by Statistical Parametric Mapping version 12 (SPM12) [17,18]; Then, the image was smoothed and normalized to the Montreal Neurological Institute (MNI) standard T1 whole brain template (standard space 91 × 109 × 91 with a resolution of 1 mm × 1 mm × 1 mm) using DARTEL normalization. After that, the image was re-oriented and spatially normalized to the template with reference to the identical anatomical position.

### 2.4. Radiomics Features Extraction

The 3D slicer v4.11.20210226 installed with PyRadiomics extension [19] was used to extract the radiomics features from the T1-weighted MPRAGE MR image. Each image in NIFTI format was loaded into the software, and the whole brain mask (91 × 109 × 91) from the Anatomical Automatic Labelling (AAL) template was loaded as the volume for feature extraction. A total of 120 radiomics features were selected to be extracted from the T1-weight MR image within the whole brain volume. The features included 19 first order statistics (FOS), 16 three-dimensional (3D) shape features (3DS), 10 two-dimensional (2D) shape features (2DS), 14 gray level dependence matrix (GLDM), 24 gray level co-occurrence matrix (GLCM), 16 gray level run length matrix (GLRLM), 16 gray level size zone matrix (GLSZM) and 5 neighboring gray tone difference matrix (NGTDM) features. The definition of features are in compliance with the description in Imaging Biomarker Standardization Initiative [20]. Details of features were listed in Table S1 of supplementary file and can be referred to the following website: https://pyradiomics.readthedocs.io/en/latest/features.html (accessed on 22 January 2022).

### 2.5. Brain Regional Volume Calculation

The volumetric segmentation and cortical thickness analysis was performed with the FreeSurfer v7.1.0 image analysis suite, which is documented and freely available for download online (http://surfer.nmr.mgh.harvard.edu/)(accessed on 22 January 2022). Brain regional volume of 45 regions were obtained and listed in Table S2 of supplementary file. The technical details of these procedures are described in prior publications [10,21,22,23,24,25,26,27,28,29,30] and in the Supplementary file.

### 2.6. Model Building and Validation

**Figure 1:**
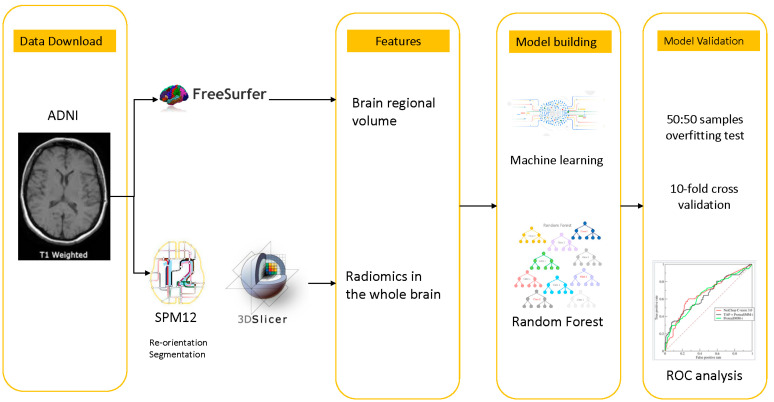
Construction process of V-model and R-model using random forest algorithm.

The obtained brain regional volumes and the radiomics features of AD, MCI and CN were used to build the model to differentiate the diseases. The brain regional volumes for the AD, MCI and CN groups were used to build the V-MCI/CN, V-MCI/AD and V-CN/AD models. The radiomics features obtained from the AD, MCI and CN groups were used to build the R-MCI/CN model, R-MCI/AD and R-CN/AD models. The random forest algorithm in the software R was employed for each model as classifier with a maximum of 500 trees. As the sampling variability came from the grouping of training dataset, validation dataset and test dataset, 70%, 15% and 15% of all data were randomly selected as training dataset, validation dataset by and testing dataset, respectively. The expected performance of the resulting model was evaluated by the 10-fold cross validation. In addition, to avoid 50 samples of each group was selected randomly for model over-fitting test. Figure 1 showed the construction process of the V-model and R-model using random forest algorithm.

### 2.7. Statistical Analysis

The model performance analysis was evaluated by the area under the receiver operating characteristics (ROC) curve (AUC). An average of AUC, accuracy, sensitivity and specificity was reported with standard deviation. SPSS version 26.0 was used to generate the ROC curves for each model.

The ROCkit (1995) version 1.1B2 was used to conduct the chi square test for the ROC curve comparison between the two models for the same group. The significance level of *p*-value of less than 0.05 was the probability that the two ROC curves are significantly different from each other.

## 3. Result

### 3.1. Overfitting Test

By selecting the same amount of sample size (*n* = 50) from each of the groups, each model was tested to avoid overfitting. All models showed AUC less than 1 and achieved similar AUC as the actual model. Details can be found in Table 1.

### 3.2. Differentiate MCI from CN

**Figure 2:**
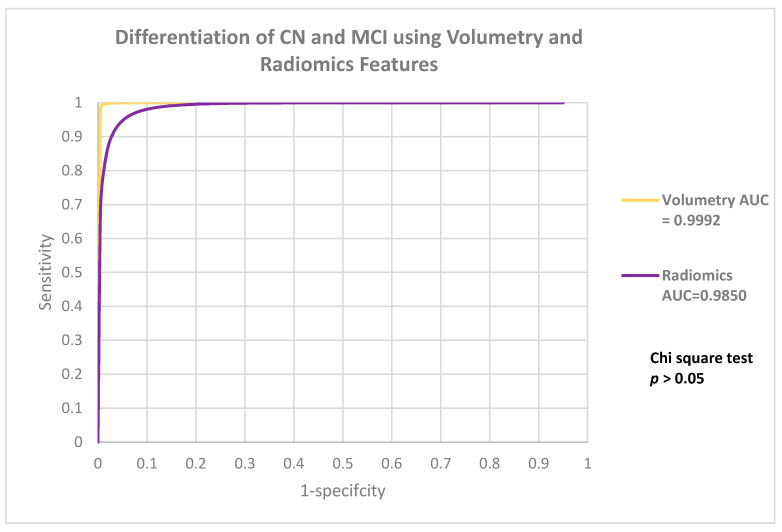
It showed the ROC comparison between the models.

The performances of V-model and R-model in classifying MCI categories were listed in Table 2. Both models achieved excellent performance, with accuracy of 0.9905 ± 0.0061 and 0.9345 ± 0.0076, respectively. Performance analysis showed that there is no significant difference between the ROC of both models (*p* = 0.056). Figure 2 showed the ROC comparison between the models.

The important variables in each models were also obtained. In the V-model, the volume of right and left inferior lateral ventricles, right and left hippocampus, right and left lateral ventricles, central, and mid-anterior corpus callosum were important variables which had high impact in prediction accuracy. In R-model, the features related to Gray Level size zone matrix (GLSZM) and Gray level Dependence Matrix (GLDM) were major categories with features that contribute to the high prediction accuracy. Details were listed in Table 3.

### 3.3. Differentiate AD from MCI

**Figure 3:**
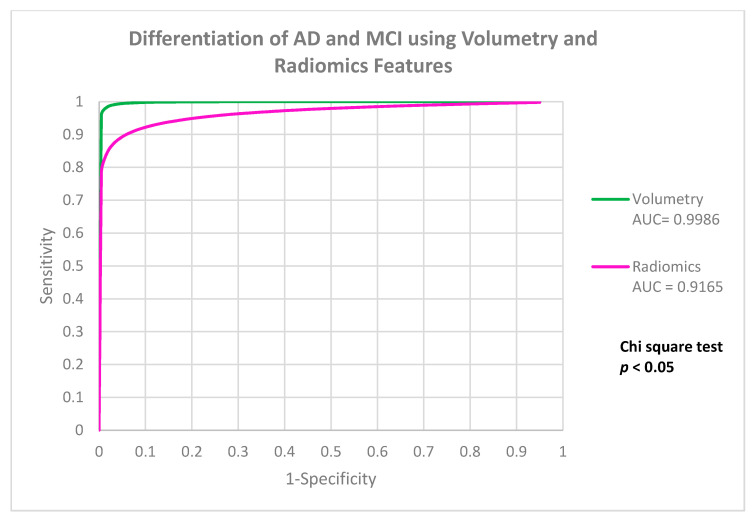
It showed the ROC comparison between the models.

The performances of V-model and R-model in classifying MCI categories were listed in Table 4. Both models achieved excellent performance, with accuracy of 0.977 ± 0.0116 in V-model, and accuracy of 0.9401 ± 0.0079 in the R-model. Performance analysis showed that there the V-model was significantly more accurate in differentiate AD from MCI, when compared to R-model with a difference of 3.69% (*p* = 0.001). Figure 3 showed the ROC comparison between the models.

The important variables in each model were also obtained. In the V-model, other than the volume of right and left hippocampus, the right and left cerebellum cortex, left and right amygdala, mid-posterior corpus callosum were important variables which had high impact in prediction accuracy. In R-model, other than the features related to GLSZM and GLDM, the features under Gray level Dependence Matrix (GLCM) had high importance in accurate prediction. Details were listed in Table 5.

### 3.4. Differentiate AD from CN

**Figure 4:**
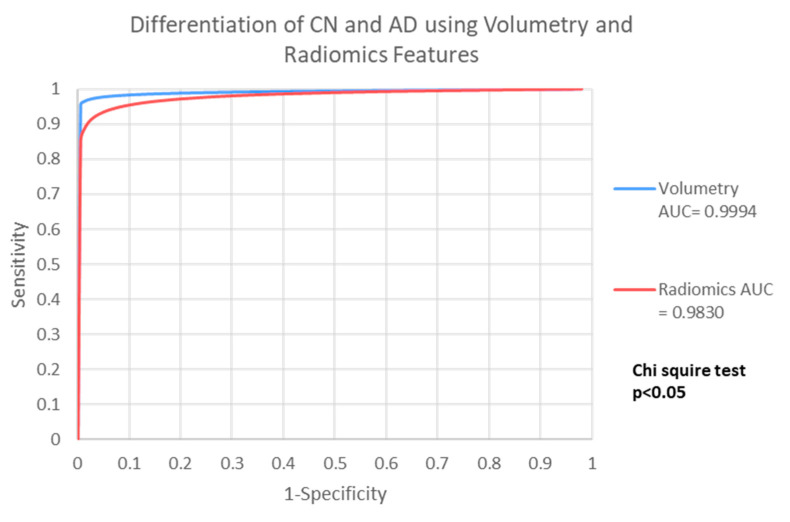
It showed the ROC comparison between the models.

The performances of V-model and R-model in classifying AD from CN were listed in Table 6. Both models achieved excellent performance, with accuracy of 0.9877 ± 0.0086 in V-model, and accuracy of 0.9292 ± 0.00169 in the R-model. Performance analysis showed that the V-model is significantly more accurate in differentiate AD from MCI, when compared to R-model with a difference of 5.85% (*p* = 0.0362). Figure 4 showed the ROC comparison between the models.

The important variables in each model were also obtained. In the V-model, other than the volume of right and left hippocampus, the right and left inferior lateral ventricles, left and right amygdala, mid-posterior corpus callosum were important variables which had high impact in prediction accuracy. In the R-model, other than the features related to GLSZM, GLDM and GLCM, the 3DS features had high importance in accurate prediction. Details were listed in Table 7.

## 4. Discussion

In this study, our proposed model based on radiomics features achieved excellent performance in differentiation of AD, MCI and CN, with comparable results obtained by traditional volumetry analysis. Further, we identified the volume of relevant brain regions, as well as radiomics features that had the potential to serve as biomarkers to differentiate MCI from CN and AD from MCI.

### 4.1. Volumetric Analysis in AD, MCI and CN

Brain regional atrophy or ventricle enlargement has long been used to differentiate AD from CN, in particular, hippocampal atrophy and enlargement of lateral ventricles. In this study, whole brain automatic segmentation was carried out by FreeSurfer, and all brain regions were included to build the V-model for disease differentiation. Through machine learning, our model selected the brain regions which are the same as those volumetric analysis studies for AD diagnosis, including left and right hippocampus, left and right amygdala and left and right inferior lateral ventricles. These brain regions were well established relevant to AD diagnosis.

However, structural change is not easily detectable in MCI when compared to CN, especially for early onset subtype, even though with the voxel-based method. This makes the application of volumetric analysis in differentiating MCI from CN very challenging. Previous studies suggested to add other biomarkers, including apolipoprotein ε4 status [31], grey matter loss [32] or functional connectivity [33] in volumetric analysis to improve the efficacy in differentiating MCI from CN. These required clinical, genetic information or multiple imaging modalities which complicate computer assisted algorithm. Other studies obtained significant difference in cortical thickness between MCI and CN when using surface-based volumetric segmentation method [34]; or to divide the corpus callosum into five equally spaced regions using surface-based method and included them as volumetric variables [35,36]. In this study, 45 brain regional volumes were segmented by surface-based method, all were used to develop the V-model. The volume of hippocampi, lateral ventricles, inferior lateral ventricles, fourth ventricle, the middle anterior and central part of corpus callosum were suggested as important variables in differentiating MCI from CN, and the model achieved an accuracy over 99%, with high sensitivity and specificity. The volume of these brain regions were previously validated by other studies [11,14,15,37,38], indicated that the variable selection by the random forest algorithm were supported by clinical observations.

### 4.2. Radiomics Features in AD, MCI and CN

While these brain regions reflect the local volume change, but not the pathological mechanism related to the CN and MCI. In this study, two models were developed based on 107 whole brain radiomics features to identify MCI from CN and AD from MCI, respectively. The model identified several GLSZM, GLDM and GLRLM features as important variables for MCI from CN. For GLSZM, they were small and large area emphasis, gray level non-uniformity normalized, low and high gray zone emphasis, and large area high gray level emphasis. GLSZM measures the number of gray level zones and their sizes, it has proven to be useful in studying neuropathological heterogeneity [39], which is a determinant of AD and early MCI [40]. For GLDM, they were dependence entropy, small dependence low gray level emphasis, and large dependence high graph level emphasis. GLDM describes the gray level dependence in an image. It corresponds to the irregular spatial distribution and heterogeneous distribution between voxels, reference to the complex structural relationship between the voxels. It also reflects the degree of association between different voxels in the whole brain [41]. Previous study suggested that early onset of AD showed more complex pattern in T1W images [42]. GLRLM relates to the coarseness and complexity, which reflect the degree of association between different pixels in the whole brain [41]. Our results were coherent with Jain et al., 2021 study, which suggested GLSZM, GLDM and GLRLM were promising features utilized in the whole brain for dementia classification [43].

For the model to differentiate AD from MCI, other than the GLSZM, GLDM and GLRLM which described previously, the joint energy and maximum probability of GLCM and kurtosis of first order statistics were included as major variables. GLCM features reflects the spatial correlation between pixel gray level values in the image [44]. It is a texture based feature which related to the construction of signatures, and indicates the brain microstructural damage [45]. White matter degeneration is an important feature of AD, where diffusion tensor imaging revealed that microstructural damage mainly in occipital and frontal region in MCI, but widespread throughout the whole brain in AD patient [46]. GLCM relates to the white matter degeneration, its extensiveness can be a major factor to differentiate AD from MCI, and AD from CN.

### 4.3. Models Performance Analysis

In this study, we reported that the well-established volume features and radiomics textural features had comparable and relevant characteristics in classifying the dementia groups. In the MCI and CN differentiation, both V-model and R-model demonstrated similar excellent performance with no significant difference. The results suggested that using either brain regional volumes or radiomics features can differentiate MCI and CN effectively. On the other hand, although the V-model had better accuracy to distinguish AD and MCI or AD and CN when compared to the R-model, both V-model and R-model achieved good accuracy, sensitivity and specificity. The radiomics model can be an alternative to volumetry as a biomarker to differentiate AD and MCI from CN.

To avoid model overfitting, equal number of samples in disease groups were selected randomly to test the model. The test results demonstrated similar AUC, accuracy, sensitivity and specificity with the actual model, suggested that the risk of overfitting was mitigated. To provide realistic model performance estimation, the ten-fold cross validation was adopted for model training and validation. Further studies are suggested to include an external dataset for validation.

### 4.4. Efficiency in Feature Extraction and Model Building

For surface-based volumetry segmentation using FreeSurfer, the run times were approximately 38 h for an AMD opertron 64 bit 2.5 GHz processor for both brain hemispheres for one patient [47]. While for radiomics features, it took less than ten minutes for 120 features retrieved by Pyradiomics. Both models were built using random forest algorithm which required less than two minutes.

Other than the software run time, the selection of regions of interest in the volumetry analysis required clinician’s expertise and experience. Using automatic segmentation in volumetry analysis and consider all brain regions for model building enables the process to become operator independent. It is exceptional useful when the model is designed to aid diagnosis, which required a clinician to confirm. Based on the clinical application, the radiomics features can be extracted efficiently, and can be loaded to the model for diagnosis prediction.

### 4.5. Limitation of Study

Compared to previous studies, our sample size was not very small. However, including other cohorts of study with independent samples may help to improve the reliability of the diagnostic models. Additionally, we used T1W MR image alone to build the diagnostic models, further investigations can be carried out by including other imaging modalities, for example 18F-flutametamol PET images or functional MRI images for model development. Besides the radiomics features extracted from the whole brain, radiomics from individual brain structures can be extracted for further investigations. In this study, the images were collected from overseas, further validation using image data from local patients is suggested.

## 5. Conclusions

This study showed that the radiomics features from T1-weighted MR images achieved excellence performance in differentiating AD, MCI and CN. Compared to the volumetry method, the accuracy, sensitivity and specificity were slightly lower in using radiomics, but still attained very good and reliable classification of the three stages of neurodegenerations. In view of the convenience and operator independence in feature extraction, radiomics can be a quantitative biomarker to differentiate the disease groups.

## Figures and Tables

**Table 1:** Overfitting test results.

Overfitting Test	MCI vs. CN Sample Size: CN = 50, MCI = 50		AD vs. MCI Sample Size: AD = 50, MCI = 50		AD vs. CN Sample Size: CN = 50, AD = 50	
	**Volumetry**	**Radiomics**	**Volumetry**	**Radiomics**	**Volumetry**	**Radiomics**
AUC	0.9988	0.9698	0.9948	0.9362	0.9788	0.98
Accuracy	0.96	0.91	0.95	0.91	0.91	0.98
Sensitivity	0.92	0.88	0.92	0.84	0.94	0.99
Specificity	0.99	0.94	0.98	0.98	0.88	0.96

**Table 2:** Models for differentiating MCI from CN.

V-Model and R-Model for MCI and CN Differentiation Sample Size MCI = 293:CN = 192		
	**Volumetry (V-Model)**	**Radiomics (R-Model)**
AUC	0.9992 ± 0.0022	0.9850 ± 0.0032
Accuracy	0.9905 ± 0.0061	0.9345 ± 0.0076
Sensitivity	0.9990 ± 0.0016	0.9671 ± 0.0143
Specificity	0.9786 ± 0.016	0.8848 ± 0.0353

**Table 3:** Important variables in V-Model and R-model to differentiate MCI from CN.

Brain Regions	Radiomics Features	
Right Inferior lateral Ventricle	GLSZM	Large Area High Gray Level Emphasis
Left Hippocampus	GLDM	Small Dependence Low Gray Level Emphasis
4th ventricle	GLDM	Large Dependence High Gray Level Emphasis
Left cerebellum cortex	GLRLM	Short Run Low Gray Level Emphasis
Right Lateral Ventricle	GLDM	Dependence Entropy
Right Hippocampus	GLSZM	Small Area Emphasis
Corpus Callosum-Central	GLSZM	Large Area Emphasis
Corpus Callosum-Mid Anterior	FOS	Median
Left lateral ventricle	GLSZM	Gray Level Non-Uniformity
Left Inferior lateral Ventricle	GLSZM	Low Gray Level Zone Emphasis

**Table 4:** Model comparison for differentiating AD from MCI.

V-Model and R-Model for MCI and AD Differentiation Sample Size MCI = 293:AD = 97		
	**Volumetry (V-Model)**	**Radiomics (R-Model)**
AUC	0.9986 ± 0.0013	0.9714 ± 0.0175
Accuracy	0.9770 ± 0.0116	0.9401 ± 0.0079
Sensitivity	0.9165 ± 0.0472	0.8010 ± 0.0327
Specificity	0.9973 ± 0.0031	0.9873 ± 0.0100

**Table 5:** Important variables in V-Model and R-model to differentiate AD from MCI.

Brain Regions	Radiomics Features	
Right Amygdala	GLSZM	Gray Level Non-Uniformity
Left Cerebellum Cortex	GLRLM	Low Gray Level Run Emphasis
Right Cerebellum Cortex	GLDM	Large Dependence Low Gray Level Emphasis
Left Amygdala	GLSZM	Low Gray Level Emphasis
Left Hippocampus	GLCM	Maximum Probability
Right Accumben Areas	GLCM	Joint Energy
Right Hippocampus	GLSZM	Zone Variance
Corpus Callosum Mid Posterior	FOS	Kurtosis
Right Pallidum	GLDM	Dependence Entropy
Right Caudate	GLSZM	High Gray Level Zone Emphasis
Left vessels	GLDM	Large Dependence High Gray Level Emphasis

**Table 6:** Model comparison for differentiating AD from CN.

V-Model and R-Model for AD and CN Differentiation Sample Size AD = 97:CN = 192		
	**Volumetry (V-Model)**	**Radiomics (R-Model)**
AUC	0.9994 ± 0.0011	0.9830 ± 0.0095
Accuracy	0.9877 ± 0.0086	0.9292 ± 0.0169
Sensitivity	0.9763 ± 0.0182	0.8967 ± 0.0416
Specificity	0.9990 ± 0.0022	0.9712 ± 0.0162

**Table 7:** Important variables in V-Model and R-model to differentiate AD from CN.

Brain Regions	Radiomics Features	
Left Hippocampus	GLSZM	Large Area High Gray Level Emphasis
Right Hippocampus	GLSZM	Small Area Emphasis
Left Inferior Lateral Ventricle	GLSZM	Size Zone Non-Uniformity Normalized
4th Ventricle	3DS	Voxel Volume
Left Amygdala	3DS	Mesh Volume
Right Pallidum	GLRLM	Short Run Low Gray Level Emphasis
Right Lateral Ventricle	3DS	Surface Area
Right Inferior Lateral Ventricle	GLDM	Large Dependence High Gray Level Emphasis
Right Amygdala	GLCM	Inverse Variance
Left Thalamus	GLCM	Sum Entropy
Mid posterior Corpus Callosum	GLDM	Small Dependence Low Gray Level Emphasis

## Data Availability

Publicly available datasets were analyzed in this study. This data can be found at: http://adni.loni.usc.edu/ (accessed on 22 January 2022). Data used in preparation of this article were obtained from the Alzheimer’s Disease Neuroimaging Initiative (ADNI) database (adni.loni.usc.edu). As such, the investigators within the ADNI contributed to the design and implementation of ADNI and/or provided data but did not participate in analysis or writing of this report. A complete listing of ADNI investigators can be found at: http://adni.loni.usc.edu/wp-content/uploads/how_to_apply/ADNI_Acknowledgement_List.pdf (accessed on 22 January 2022). Approval was obtained on 28 February 2022 from the ADNI Data and Publications Committee (DPC) for the use of ADNI data for publication.

## Supplementary Materials

The following supporting information can be downloaded at: https://www.mdpi.com/article/10.3390/life12040514/s1, Table S1: 107 Radiomics features extracted from T1 MPRAGE image by 3D slicer; Table S2: 45 Brain regions retrieved from FreeSurfer.
