# Supplementary material for: Radiomics-Based Artificial Intelligence Differentiation of Neurodegenerative Diseases with Reference to the Volumetry
Source: Life (Basel). 2022 Mar 31;12(4):514. doi: 10.3390/life12040514 (PMC9024778; doi:10.3390/life12040514)
Supplement: Supplementary file 1 [file life-12-00514-s001.zip › life-1638819-supplementary.pdf]

life

MDPI

Supplementary

Radiomics-Based Artificial Intelligence Differentiation of Neurodegenerative Diseases with Reference to the Volumetry

Eva Y W Cheung [1], Anson C M Chau [2], Fuk Hay Tang [1,*], for the Alzheimer's Disease Neuroimaging Initiative **

[1] School of Medical Health and Sciences, Tung Wah College, 19/F, 31 Wylie Road, Ho Man Tin, Hong Kong, China; cheungevayw@gmail.com, evacheung@twc.edu.hk
[2] Medical Radiation Science, Allied Health and Human Performance Unit, University of South Australia, City East Campus, Bonython Jubilee Building, 1-26, Adelaide, SA 5001, Australia anson.chau@unisa.edu.au
* Correspondence: fhtang@twc.edu.hk

Citation: Cheung, E.Y.W.; Chau, A.C.M.; Tang, F.H.; on behalf of the Alzheimer's Disease Neuroimaging Initiative. Radiomics-Based Artificial Intelligence Differentiation of Neurodegenerative Diseases with Reference to the Volumetry. *Life* **2022**, *12*, 514. https://doi.org/10.3390/life12040514

Academic Editors: Christopher Lai and Suchart Kothan

Received: 28 February 2022
Accepted: 28 March 2022
Published: 31 March 2022

Publisher's Note: MDPI stays neutral with regard to jurisdictional claims in published maps and institutional affiliations.

Copyright: © 2022 by the authors. Licensee MDPI, Basel, Switzerland. This article is an open access article distributed under the terms and conditions of the Creative Commons Attribution (CC BY) license (https://creativecommons.org/licenses/by/4.0/).

Image acquisition parameters

The MR T1-weighted structural images were acquired using Siemens scanner, using a 3D MPRAGE (magnetization prepared rapid gradient-echo imaging) sequence. The scanning parameters are as follows: repetition *time* (TR) = 2,300 ms, matrix = 240 × 256 × 176, slice thickness = 1.2 mm, and those parameters in General Electric scanner were as follows: TR = 7 ms, matrix = 256 × 256 × 166, slice thickness = 1.2 mm and those parameters in Philips scanner were as follows: TR = 6.8 ms, matrix = 256 × 256 × 170, slice thickness = 1.2 mm, respectively. More detailed information about the image acquisition procedures is available on the ADNI website.

Technical details of brain regional volume calculation by Freesurfer

Briefly, this processing includes motion correction and averaging [29] of multiple volumetric T1 weighted images (when more than one is available), removal of non-brain tissue using a hybrid watershed/surface deformation procedure[31], automated Talairach transformation, segmentation of the subcortical white matter and deep gray matter volumetric structures (including hippocampus, amygdala, caudate, putamen, ventricles) [10,24] intensity normalization [32], tessellation of the gray matter white matter boundary, automated topology correction [23,33] , and surface deformation following intensity gradients to optimally place the gray/white and gray/cerebrospinal fluid borders at the location where the greatest shift in intensity defines the transition to the other tissue class [21,22,34]. Once the cortical models are complete, a number of deformable procedures can be performed for further data processing and analysis including surface inflation [25], registration to a spherical atlas which is based on individual cortical folding patterns to match cortical geometry across subjects [26], parcellation of the cerebral cortex into units with respect to gyral and sulcal structure [35,36], and creation of a variety of surface based data including maps of curvature and sulcal depth. This method uses both intensity and continuity information from the entire three dimensional MR volume in segmentation and deformation procedures to produce representations of cortical thickness, calculated as the closest distance from the gray/white boundary to the gray/CSF boundary at each vertex on the tessellated surface [22]. The maps are created using spatial intensity gradients across tissue classes and are therefore not simply reliant on absolute signal intensity. The maps produced are not restricted to the voxel resolution of the original data thus are capable of detecting submillimeter differences between groups. Procedures for the measurement of cortical thickness have been validated against histological analysis[37] and manual measurements [38,39]. Freesurfer morphometric procedures have been demonstrated to show good test-retest reliability across scanner manufacturers and across field strengths [27,30]

Table S1. 107 Radiomics features extracted from T1 MPRAGE image by 3D slicer.

	**First order statistics (19) (FOS)**	**3D Shape Features (16) (3DS)**	**2D Shape Features (10) (2DS)**	**Grey Level Co-occurrence Matrix (24) (GLCM)**	**Grey Level Size zone Matrix (16) (GLSZM)**	**Grey Level Run Length Matrix (16) (GLRLM)**	**Neighboring Gray Tone Difference Matrix (5) (NGTDM)**	**Gray level Dependence Matrix (14) (GLDM)**
1	Energy	Mesh Volume	Mesh Surface	Autocorrelation	Small area emphasis	Short Run emphasis	Coarseness	Small dependence emphasis
2	Total Energy	Voxel Volume	Pixel Surface	Joint Average	Large area emphasis	Long Run emphasis	Contrast	Large dependence emphasis
3	Entropy	Surface Area	Perimeter	Cluster Prominence	Gray level non-uniformity	Gray level non-uniformity	Busyness	Gray level non-uniformity
4	Minimum	Surface Area to Volume Ratio	Perimeter to surface ratio	Cluster Shade	Gray level non-uniformity Normalized	Gray level non-uniformity Normalized	Complexity	Dependence non-uniformity
5	10th percentile	Sphericity	Sphericity	Cluster Tendency	Size Zone non-uniformity	Run Length non-uniformity	Strength	Dependence non-uniformity Normalized
6	90th percentile	Compactness1	Spherical Disproportion	Contrast	Size Zone non-uniformity Normalized	Run Length non-uniformity Normalized		Gray Level variance
7	Maximum	Compactness2	Maximum 2D diameter	Correlation	Zone percentage	Run percentage		Dependence variance
8	Mean	Spherical Disproportion	Major axis length	Difference Average	Gray level Variance	Gray level Variance		Dependence entropy b
9	Median	Maximum 3D diameter	Minor axis length	Difference entropy	Zone Variance	Run Variance		Low Gray level Emphasis
10	Inter Quartile range feature value	Maximum 2D diameter - slice	Elongation	Difference Variance	Zone Entropy	Run Entropy		High Gray level Emphasis

11	range feature value	Maximum 3D diameter - column		Joint Energy	Low Gray level Zone Emphasis b	Low Gray level Run Emphasis		Small dependence Low Gray level Emphasis
12	Mean Absolute deviation	Maximum 3D diameter - row		Joint Entropy	High Gray level Zone Emphasis	High Gray level Run Emphasis		Small dependence High Gray level Emphasis
13	Robust Mean Absolute deviation	Major axis length		Information Measure of correlation 1	Small area Low Gray level Emphasis	Short Run Low Gray level Emphasis		Large dependence Low Gray level Emphasis
14	Root Mean Squared	Minor axis length		Information Measure of correlation 2	Small area High Gray level Emphasis	Short Run High Gray level Emphasis		Large dependence High Gray level Emphasis b
15	Standard deviation	Least axis length		Inverse difference moment	Large area Low Gray level Emphasis	Long Run Low Gray level Emphasis		
16	Skewness	Elongation		Maximal correlation coefficient	Large area High Gray level Emphasis	Long Run High Gray level Emphasis		
17	Kurtosis	Flatness		Inverse difference moment normalized				
18	Variance			Inverse difference				
19	Uniformity			Inverse difference normalized				
20				Inverse variance				
21				Maximum probability				
22				Sum average				
23				Sum entropy				
24				Sum of squares				

Table S2. 45 Brain regions retrieved from FreeSurfer.

**Left-Lateral-Ventricle**	**4th-Ventricle**	**Right-Lateral-Ventricle**	**Right-Amygdala**	**non-WM-hypointensities**
Left-Inf-Lat-Vent	Brain-Stem	Right-Inf-Lat-Vent	Right-Accumbens-area	Left-non-WM-hypointensities
Left-Cerebellum-White-Matter	Left-Hippocampus b	Right-Cerebellum-White-Matter	Right-VentralDC	Right-non-WM-hypointensities
Left-Cerebellum-Cortex b	Left-Amygdala	Right-Cerebellum-Cortex	Right-vessel	Optic-Chiasm
Left-Thalamus	CSF	Right-Thalamus	Right-choroid-plexus	CC_Posterior
Left-Caudate	Left-Accumbens-area	Right-Caudate	5th-Ventricle	CC_Mid_Posterior
Left-Putamen	Left-Ventral DC	Right-Putamen	WM-hypointensities	CC_Central
Left-Pallidum	Left-vessel	Right-Pallidum b	Left-WM-hypointensities	CC_Mid_Anterior
3rd-Ventricle	Left-choroid-plexus	Right-Hippocampus b	Right-WM-hypointensities	CC_Anterior

**Data used in preparation of this article were obtained from the Alzheimer's Disease Neuroimaging Initiative (ADNI) database (adni.loni.usc.edu). As such, the investigators within the ADNI contributed to the design and implementation of ADNI and/or provided data but did not participate in analysis or writing of this report. A complete listing of ADNI investigators can be found at: http://adni.loni.usc.edu/wp-content/uploads/how_to_apply/ADNI_Acknowledgement_List.pdf.

Acknowledgement

Data collection and sharing for this project was funded by the Alzheimer's Disease Neuroimaging Initiative (ADNI) (National Institutes of Health Grant U01 AG024904) and DOD ADNI (Department of Defense award number W81XWH-12-2-0012). ADNI is funded by the National Institute on Aging, the National Institute of Biomedical Imaging and Bioengineering, and through generous contributions from the following: AbbVie, Alzheimer's Association; Alzheimer's Drug Discovery Foundation; Araclon Biotech; BioClinica, Inc.; Biogen; Bristol-Myers Squibb Company; CereSpir, Inc.; Cogstate; Eisai Inc.; Elan Pharmaceuticals, Inc.; Eli Lilly and Company; EuroImmun; F. Hoffmann-La Roche Ltd and its affiliated company Genentech, Inc.; Fujirebio; GE Healthcare; IXICO Ltd.; Janssen Alzheimer Immunotherapy Research & Development, LLC.; Johnson & Johnson Pharmaceutical Research & Development LLC.; Lumosity; Lundbeck; Merck & Co., Inc.; Meso Scale Diagnostics, LLC.; NeuroRx Research; Neurotrack Technologies; Novartis Pharmaceuticals Corporation; Pfizer Inc.; Piramal Imaging; Servier; Takeda Pharmaceutical Company; and Transition Therapeutics. The Canadian Institutes of Health Research is providing funds to support ADNI clinical sites in Canada. Private sector contributions are facilitated by the Foundation for the National Institutes of Health (www.fnih.org). The grantee organization is the Northern California Institute for Research and Education, and the study is coordinated by the Alzheimer's Therapeutic Research Institute at the University of Southern California. ADNI data are disseminated by the Laboratory for Neuro Imaging at the University of Southern California.
